# Deoxynivalenol Impairs Hepatic and Intestinal Gene Expression of Selected Oxidative Stress, Tight Junction and Inflammation Proteins in Broiler Chickens, but Addition of an Adsorbing Agent Shifts the Effects to the Distal Parts of the Small Intestine

**DOI:** 10.1371/journal.pone.0069014

**Published:** 2013-07-26

**Authors:** Ann Osselaere, Regiane Santos, Veerle Hautekiet, Patrick De Backer, Koen Chiers, Richard Ducatelle, Siska Croubels

**Affiliations:** 1 Department of Pharmacology, Toxicology and Biochemistry, Faculty of Veterinary Medicine, Ghent University, Merelbeke, Belgium; 2 Division Toxicology – Veterinary Pharmacology, Pharmacotherapy and Toxicology, Faculty of Veterinary Medicine, Utrecht University, Utrecht, The Netherlands; 3 Sanluc International NV, Gijzenzele, Belgium; 4 Department of Pathology, Bacteriology and Avian Diseases, Faculty of Veterinary Medicine, Ghent University, Merelbeke, Belgium; Catalan Institute for Water Research (ICRA), Spain

## Abstract

Broiler chickens are rather resistant to deoxynivalenol and thus, clinical signs are rarely seen. However, effects of subclinical concentrations of deoxynivalenol on both the intestine and the liver are less frequently studied at the molecular level. During our study, we investigated the effects of three weeks of feeding deoxynivalenol on the gut wall morphology, intestinal barrier function and inflammation in broiler chickens. In addition, oxidative stress was evaluated in both the liver and intestine. Besides, the effect of a clay-based mycotoxin adsorbing agent on these different aspects was also studied. Our results show that feeding deoxynivalenol affects the gut wall morphology both in duodenum and jejenum of broiler chickens. A qRT-PCR analysis revealed that deoxynivalenol acts in a very specific way on the intestinal barrier, since only an up-regulation in mRNA expression of claudin 5 in jejunum was observed, while no effects were seen on claudin 1, zona occludens 1 and 2. Addition of an adsorbing agent resulted in an up-regulation of all the investigated genes coding for the intestinal barrier in the ileum. Up-regulation of Toll-like receptor 4 and two markers of oxidative stress (heme-oxigenase or HMOX and xanthine oxidoreductase or XOR) were mainly seen in the jejunum and to a lesser extent in the ileum in response to deoxynivalenol, while in combination with an adsorbing agent main effect was seen in the ileum. These results suggest that an adsorbing agent may lead to higher concentrations of deoxynivalenol in the more distal parts of the small intestine. In the liver, XOR was up-regulated due to DON exposure. HMOX and HIF-1α (hypoxia-inducible factor 1α) were down-regulated due to feeding DON but also due to feeding the adsorbing agent alone or in combination with DON.

## Introduction

Mycotoxin contamination can occur in all agricultural commodities in the field and/or during storage, if the conditions are favorable for fungi growth [1]. Deoxynivalenol (DON), also called vomitoxin, is a trichothecene mycotoxin which is highly prevalent in Europe [2]–[4]. In poultry, DON rarely causes acute mycotoxicosis. However, chronic exposure to the toxin can lead to reduced production and an altered immune function [5]. As poultry seems to be less susceptible to DON-mycotoxicosis compared to other animals, infected cereal batches are sometimes diverted to the poultry feed production [6]. Mycotoxin-detoxifying agents are frequently used feed additives to reduce the adverse effects of mycotoxins. Detoxifiers based on clay minerals are classified by the European Food Safety Authority (EFSA) as adsorbing agents [7].

Mycotoxins are food and feed contaminants and thus after ingestion the intestine can be exposed to high concentrations of the toxins [8], [9]. The epithelial surface of the intestine is characterized by a large contact area for absorption of nutrients and xenobiotics. This surface consists of a simple columnar epithelium, which is increased by the presence of villi [10]. Both toxins and mycotoxin detoxifiers can interact with this surface area, resulting in altered extent and rate of absorption of xenobiotics such as drugs and mycotoxins. For example, we found in a previous study higher plasma concentrations of DON in animals fed contaminated feed in combination with a clay-based adsorbing agent compared to animals fed DON contaminated feed only [11], [12].

The absorbing epithelial cells (enterocytes) are connected strongly by tight junction proteins. These tight junctions seal off the luminal end of the intercellular space and so transport by this paracellular route is very limited [13].

Claudins are transmembrane proteins which form the backbone of the tight junction strands. Claudin 1 and 5 are known to interact and are important to guarantee the intestinal barrier function. Both claudins have already been characterized in chickens [14]–[16]. The family of zona occludens, including zona occludens 1 (ZO 1) and zona occludens 2 (ZO 2), is a group of scaffolding proteins which is part of the cytoplasmic plaque of the tight junctions.

The intestinal epithelial cells also contribute to the regulation of inflammatory conditions and create a kind of barrier against invading pathogens. Toll-like receptors (TLR) in the intestinal epithelium, particularly TLR4, serve as rapid pathogen sensors.

After intestinal absorption of mycotoxins these compounds reach the liver as the gateway of the portal blood draining the gastrointestinal tract. Both intestine and liver consist of rapidly proliferating cells and have a high protein turnover rate. Therefore, we may suppose that these organs are more sensitive for the action of DON [17].

The toxicity of DON is mediated by various mechanisms. Trichothecenes are potent inhibitors of the RNA, DNA and protein synthesis [18]. In addition, DON may induce the production of free radicals and cellular oxidative stress. It has been shown that oxidative stress causes up-regulation of hypoxia-inducible factor 1, subunit alpha (HIF-1α) [19], a transcription factor which regulates genes involved in inflammation and cell death [20]. Heme-oxigenase (HMOX) is another sensitive marker of oxidative injury, which affords protection against hepatocyte death [21]. Both HIF-1α and HMOX have already been characterized in chickens [22], [23]. Xanthine oxidoreductase (XOR) is an enzyme associated with the synthesis of reactive oxygen species and is part of the cellular defense enzyme systems [24]. In broilers, this enzyme is mainly expressed in the liver, but also in the intestine (60% of the amount in the liver) and other organs but in a lower amount [25]. The intestine requires an efficient immune defense at the epithelial surface, and among other factors, XOR is secreted by the enterocytes of the small intestine [26].

The aim of our study was to assess the effects of three weeks dietary exposure to DON on the small intestine and liver in broiler chickens. To this end qRT-PCR analyses were conducted to study if genes coding for oxidative stress and inflammation response are influenced by DON, both in the liver and the small intestine. In addition, the effects of DON on the intestinal morphology and intestinal barrier function were investigated with histopathology and qRT-PCR analysis, respectively. To our knowledge, this is the first *in vivo* study which observes these parameters in broiler chickens. Finally, the effects of a clay-based mycotoxin-detoxifying agent were also investigated during our trial.

## Materials and Methods

### Ethics Statement

The protocol was approved by the Ethical Committee of the Faculty of Veterinary Medicine (Ghent University) (EC 2010/064 and EC 2010/076). All husbandry practices and euthanasia were performed with full consideration of animal welfare.

### Animals and Diets

The animals and the experimental design have been described elsewhere [12]. In brief, 32 1-day-old broiler chickens were fed uncontaminated feed during an acclimatization period of ten days. Afterwards, the animals were divided into four different dietary groups of 8 animals each: a control group receiving uncontaminated feed, a group receiving uncontaminated feed+adsorbing agent, a third group receiving naturally DON contaminated feed and a group fed naturally DON contaminated feed+adsorbing agent. Analyses of the feed were performed by a multi-mycotoxin LC-MS/MS method [3]. The naturally contaminated feed was contaminated as follows: DON (7.540±2.20 mg/kg), 3-acetylDON (1.481±0.57 mg/kg), fumonisin B1 (0.700±0.08 mg/kg), fumonisin B2 (0.201±0.02 mg/kg) and fumonisin B3 (0.207±0.08 mg/kg). The adsorbing agent (illite-ambrosite clay) was added in a concentration of 1.5 kg/ton feed. After three weeks of feeding, the animals were euthanized and liver and intestinal samples were immediately collected. From the small intestine, samples were taken at three different locations: 2 cm after the gizzard (duodenum), just before Meckel’s diverticulum (jejunum) and two cm before the ileo-cecal transition (ileum). Intestinal and liver samples were rinsed in phosphate buffered saline (PBS). Afterwards, the samples for qRT-PCR analysis were immediately frozen in liquid nitrogen and stored at −80°C until analysis. Samples for morphological examination were also rinsed in PBS and then fixed in 4% (v/v) phosphate buffered formalin.

### Quantitative RT-PCR Method to Analyze the Intestinal Barrier Function, Inflammation and Oxidative Stress

RNA from samples of liver and intestine (duodenum, jejunum and ileum) were isolated using the SV Total RNA Isolation System (Promega, Madison, WI, USA) according to the manufacturer’s instructions, and total RNA was quantified by spectrophotometry (Nanodrop ND-1000, Thermo Scientific, Wilmington, NC, USA). Subsequently, 1 µg of extracted total RNA was reverse transcribed with the iScriptTM cDNA Synthesis kit (Biorad, Hercules, CA, USA). The obtained cDNA was diluted to a final concentration of 30 ng/µL. Primers were commercially produced (Eurogentec, Nijmegen, the Netherlands) (Table 1). The primers used were selected based on specificity and efficiency by qPCR analysis of a dilution series of pooled cDNA at a temperature gradient (55°C to 65°C) for primer-annealing and subsequent melting curve analysis. The reaction mixture for the qPCR containing 10 µL of the diluted cDNA was mixed with 15 µL iQSYBR Green Supermix (Biorad), forward and reverse primers (final concentration of 0.4 pmol/µL for each primer) and sterile water according to the manufacturer's instructions. qPCR was performed using the MyiQ single-colour real-time PCR detection system (Biorad) and MyiQ System Software Version 1.0.410 (Biorad). Amplification efficiency was determined per plate using linregPCR. Data were analyzed using the efficiency corrected Delta-Delta-Ct method [27]. Housekeeping genes were tested for all the test conditions after which most stable housekeeping genes for liver and intestinal samples were selected using the geNorm software (data not shown). The most stable housekeeping genes had a M-value between 0.2 and 0.5. To determine if the inclusion of an additional housekeeping gene was required, the cut-off value for variation was set at 0.2. The fold-change values of the genes of interest (GOIs) were normalized using two housekeeping genes: hypoxanthine-guanine phosphoribosyl transferase (HPRT) and hexose-6-phosphate dehydrogenase (H6PD). The mRNA expression of proteins involved in oxidative stress i.e. HMOX, HIF-1α and XOR were evaluated in the liver and intestine. Furthermore, mRNA expression of the tight junctions proteins claudin 1 and 5 (CLDN1 and CLDN5) and zona occludens 1 and 2 (ZO1 and ZO2) in sections from the duodenum, jejunum and ileum were measured. Two compounds of the immune system, namely Toll-like receptors (TLR) 2 and 4 were also investigated during our study. For the validation of the qPCR assays following criteria were applied: slope between −3.6 and −3.1, efficiency between 90 and 110%, R^2^>0.99.

**Table 1:** Primers used for the quantification of housekeeping genes (HKG) and genes of interest (GOI).

Gene	Accession N°	Primer	Sequence	Product size (pb)	Annealing T°
HKG					
HPRT	NM_204848.1	Forward	5′ CGTTGCTGTCTCTACTTAAGCAG 3′	90	65
		Reverse	5′ GATATCCCACACTTCGAGGAG 3′		
H6PD	XM_425746.2	Forward	5′ GGAGAACCAGCACTTCTTAGAC 3′	84	64
		Reverse	5′ GGGTTCAGCAATTCCACTG 3′		
GOI					
CLDN1	NM_001013611	Forward	5′ CTGATTGCTTCCAACCAG 3′	140	57–59
		Reverse	5′ CAGGTCAAACAGAGGTACAAG 3′		
CLDN5	NM_204201	Forward	5′ CATCACTTCTCCTTCGTCAGC 3′	111	56–65
		Reverse	5′ GCACAAAGATCTCCCAGGTC 3′		
HIF-1α	NM_204297	Forward	5′ CACCATTACCATACTTCAGCAG 3′	88	65
		Reverse	5′ CTTCACATCATCCACACGTTC 3′		
HMOX	NM_205344	Forward	5′ CTTGGCACAAGGAGTGTTAAC 3′	78	61–63
		Reverse	5′ CATCCTGCTTGTCCTCTCAC 3′		
TLR2	NM_204278	Forward	5′ CCTGCAACGGTCATCTCAG 3′	135	59
		Reverse	5′ GTCTCAGGGCTTGTTCTTCAG 3′		
TLR4	NM_001030693	Forward	5′ CTGACCTACCCATCGGACAC 3′	111	59
		Reverse	5′ GCCTGAGAGAGGTCAGGTTG 3′		
XOR	NM_205127	Forward	5′ GTGTCGGTGTACAGGATACAGAC 3′	110	61
		Reverse	5′ CCTTACTATGACAGCATCCAGTG 3′		
ZO1	XM_413773	Forward	5′ CTTCAGGTGTTTCTCTTCCTCCTC 3′	131	59
		Reverse	5′ CTGTGGTTTCATGGCTGGATC 3′		
ZO2	NM_204918	Forward	5′ CGGCAGCTATCAGACCACTC 3′	87	64–65
		Reverse	5′ CACAGACCAGCAAGCCTACAG 3′		

### Morphological Examination of the Gut Wall

Formalin-fixed intestinal samples were dehydrated in xylene and embedded in paraffin. With a microtome (Microm, Prosan, Merelbeke, Belgium), sections of 4 µm thickness were cut and mounted in glass slides. Afterwards, deparaffination occurred in xylene (2 times 5 min) and then rehydratation occurred in isopropylene (5 min), 95% alcohol (5 min) and 50% alcohol (5 min). Sections were stained with haematoxylin and eosin. Using light microscopy, villus height and crypt depth (10 villi per intestinal segment) from each of 8 chickens per treatment, were measured. For this, a Leica Camera DFC320 (Leica Microsystems Ltd, Wetzlar, Germany) coupled to a computer-based image analysis system LAS v.3.8. (Leica Microsystems Ltd) was used. Only intact villi were measured. Measurements were done on cross-sections of ring-shaped intestinal segments.

### Data Analysis

Results were compared by ANOVA after determination of normality and variance homogeneity. Multiple comparisons were performed using a LSD post-hoc test. Not normally distributed data were analyzed using the non-parametric Kruskal-Wallis analysis, followed by a Mann-Whitney test using SPSS 19.0 Software (SPSS Inc., Chicago, IL, USA). A *P*-value of <0.05 was considered statistically significant.

## Results

### Not only DON but also the Adsorbing Agent Alters mRNA Expression of Oxidative Stress Markers in Liver of Broiler Chickens

**Figure 1:**
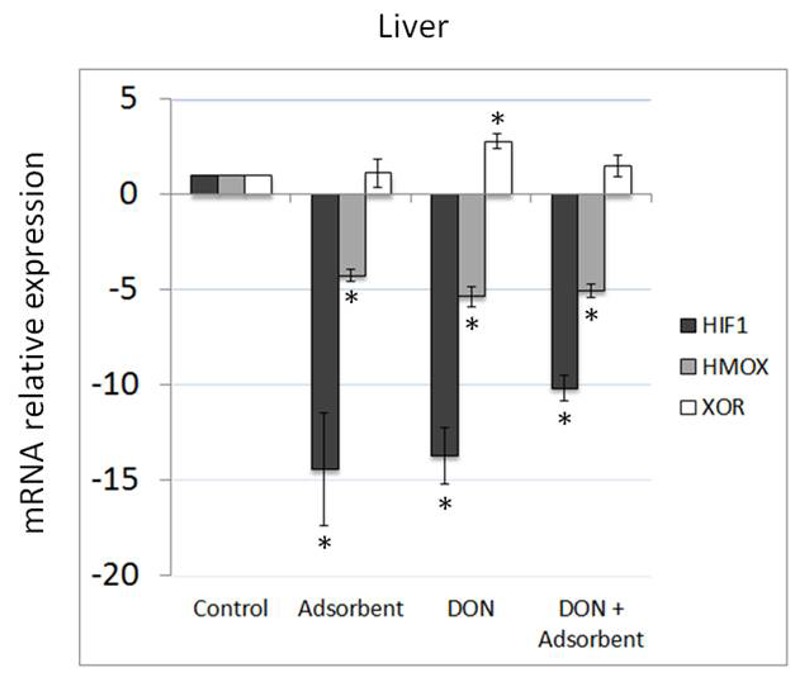
Effects of DON and an adsorbent on oxidative stress in the liver of broiler chickens. Results are presented as mean (± SEM) mRNA expression. Fold change in gene expression levels of the chicken liver relative to control group, which is considered 1. * Indicates significant differences between treated and control animals.

In the liver, both HIF-1α and HMOX mRNA were significantly down-regulated for all the broiler chickens receiving either DON, an adsorbing agent or DON and the adsorbing agent, when compared to the control group. Differently, XOR was significantly up-regulated in the group receiving the DON contaminated feed. The group receiving an adsorbing agent, whether or not in combination with DON contaminated feed was not affected. Data are shown in Figure 1.

### DON Leads to Oxidative Stress in the Jejunum and in the Ileum of Broiler Chickens in Combination with an Adsorbing Agent

**Figure 2:**
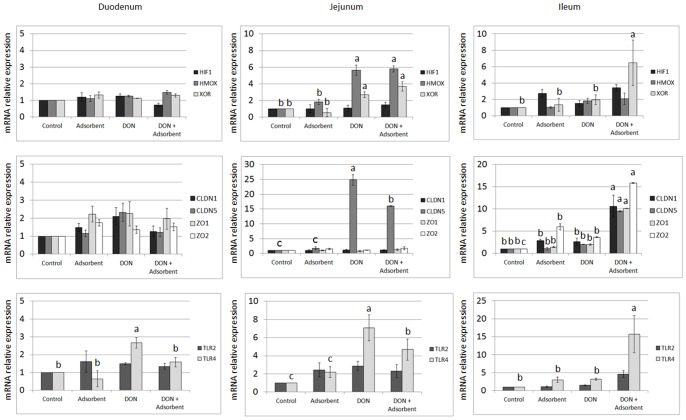
Effects of DON and an adsorbent on intestinal barrier in broiler chickens. Results are presented as mean (± SEM) mRNA expression. Fold change in gene expression levels of the chicken intestines relative to control group, which is considered 1. ^a–c^ Different lower-case letters indicate significant differences between groups.

For the small intestine, the expression of HIF-1α, HMOX and XOR mRNA was investigated in the duodenum, jejunum and ileum. Expression of HIF-1α was unaltered in the intestine, independently on the treatment or intestinal section. On the other hand, HMOX and XOR were significantly up-regulated in the jejunum of animals fed the DON contaminated feed, independently on the supplementation of an adsorbing agent. For the last part of the small intestine, the ileum, only XOR was up-regulated when animals were fed with feed containing DON and the adsorbing agent (Figure 2).

### DON and Adsorbent do not Affect Duodenal Barrier Function, but do so in Jejunum and Ileum

As observed for oxidative stress markers, barrier function of duodenum was unaffected by both DON and adsorbing agent, while jejunum presented a significant up-regulation of CLDN5 mRNA when animals were fed with DON contaminated feed. Feed supplementation with the adsorbing agent did significantly reduce the CLDN5 mRNA expression when compared to DON, but its expression remained significant higher than that observed in the control. The strongest effect on tight junctions was observed in the ileum when animals were fed with feed contaminated with DON and supplemented with the adsorbing agent, with a significant up-regulation of CLDN1, CLDN5, ZO1 and ZO2 mRNA (Figure 2).

### DON Leads to Inflammatory Reaction in Duodenum and Jejunum, but its Negative Effect in the Ileum Depends on the Feed Supplementation with an Adsorbing Agent

A significant up-regulation of TLR4 mRNA was observed in the duodenum and jejunum of animals fed with DON contaminated feed. Although feed supplementation with an adsorbing agent was efficient to decrease the TLR4 expression, it was efficient to recover control levels only in the duodenum and not in the jejunum. Finally, as observed with tight junctions’ analysis, ileum exposure to DON and adsorbing agent resulted in the significant up-regulation of TLR4 mRNA (Figure 2).

### DON Alters the Gut Wall Morphology in Duodenum and Jejunum of Broiler Chickens, but Addition of an Adsorbing Agent Counteracts these Effects

Feeding DON contaminated feed resulted in a decreased villus length and crypt depth both in duodenum and jejunum of the broiler chickens. Addition of an adsorbing agent resulted in longer villi, even in combination with DON and this over the entire length of the small intestine. The crypt depth however, was not influenced by the addition of an adsorbing agent to control feed in the duodenum and jejunum, when compared to the control group. On the other hand, the adsorbing agent had a positive effect on the crypt depth in these intestinal parts, when added to DON contaminated feed. In the ileum, no effect of feeding DON contaminated feed without an adsorbing agent was observed. In this part of the small intestine, it was the adsorbing agent in combination with DON or not which resulted in higher villi and deeper crypts (Table 2).

**Table 2:** Length of villi (µm) and crypt depth (µm) in duodenum, jejunum and ileum after 3 weeks feeding a control diet or feed contaminated with DON, either or not supplemented with an adsorbing agent. Results are presented as mean values and standard deviations of fifteen villi or crypts measured from 8 chickens per treatment group.

	Control	Adsorbingagent	DON	DON+adsorbing agent
Duodenum				
*Villus height*	1734±26^a^	1773±43^c^	1449±31^b^	1789±39^c^
*Crypt depth*	131±7^a^	134±7^a^	114±9^b^	128±8^a^
Jejunum				
*Villus height*	1343±37^a^	1521±39^c^	1184±48^b^	1509±43^c^
*Crypt depth*	120±8^a^	116±10^a^	101±8^b^	109±7^c^
Ileum				
*Villus height*	596±30^a^	773±63^b^	616±38^a^	744±63^b^
*Crypt depth*	113±6^a^	124±17^b^	110±15^a^	119±18^ab^

a,b mean values within a row with unlike superscript letters are significantly different (p≤0.05).

## Discussion

Being an interface between the outside world and the inside body, the gastro-intestinal tract (GIT) is a dynamic barrier [28]. This barrier is responsible for two major processes, which are on the one hand uptake of nutrients and fluids and on the other hand defense mechanism against xenobiotics. We performed a study with broiler chickens fed with naturally contaminated feed to investigate the effects of DON on the intestinal barrier and hepatic function. Co-contamination of different mycotoxins in naturally contaminated feed is common and this was also the case for the experimental feed used in this study. DON was the most prevalent mycotoxin and was even present in a concentration higher than the European recommended maximum level of 5 mg/kg [29]. The other contaminant present, 3-acetylDON, is considered a masked mycotoxin, i.e. a conjugated form of DON also produced by *Fusarium* fungi. It is hypothesized that this conjugated form may be hydrolysed and release DON *in vivo*, but the question remains whether this occurs in every animal species and if this occurs already in the GIT and/or liver and/or systemic circulation. The sum of the concentration of the co-contaminants fumonisin B1 and B2 of 0.901 mg/kg was much lower than the European guidance value of 20 mg/kg in poultry feed [29]. Thus, the co-contamination with fumonisins can be considered as negligible. In our study, three weeks feeding DON at 7.54 mg/kg feed reduced the villus height and the crypt depth both in the duodenum and the jejunum. Reduced villi in the duodenal and jejunal segment of the small intestine were also observed in broiler chickens after 6 weeks feeding a diet of 10 mg/kg DON [30]. Yunus et al. (2012) observed a linear correlation between increasing levels of DON and the decrease in villus height in both the mid-duodenum and mid-jejunum [31]. Possible explanation for these histological changes can be a direct irritant effect of the mycotoxin or suppression of mitosis or protein synthesis [18], [31]. In order to maintain an effective barrier function, the intestinal epithelium needs to regenerate continuously. Mature cells migrate along the crypt-villus axis towards the villus-top, in the mean time these cells become differentiated cells [32]. DON can be responsible for a reduced cell proliferation [33]–[35]. This can be an explanation for the reduced crypt depth observed during our trial. A decreased crypt depth in the mid-duodenum in broiler after chronic exposure to DON (12 mg/kg) has been reported earlier [31]. Interestingly, the adsorbing agent resulted in longer villi over the entire length of the small intestine. These longer villi seen in our study in the chickens receiving the adsorbing agent, can be responsible for the higher oral bioavailabilities of xenobiotics as observed in our previous study [12].

Several studies both *in vitro* and *in vivo* already reported that DON is able to alter intestinal permeability. Intestinal physiology can even be affected by DON in the absence of clinical signs [36]. The function of the tight junctions can be evaluated by measurements of the transepithelial electrical resistance (TEER) and of the paracellular efflux of macromolecules [37]. These techniques, however, do not give information which specific protein of the tight junctions is affected [38]. Therefore, a qRT-PCR method was applied in our study to evaluate the effects of DON on the different specific proteins of the tight junctions, namely CLDN1, CLDN5, ZO1 and ZO2. An important advantage of this technique is the generation of quantitative results, which makes it possible to detect small differences which could otherwise be missed when using immunofluorescence. Moreover, due to the lack of suitable commercial avian antibodies, no effects at the protein level could be studied. This general lack in anti-chicken antibodies for use in Western blot and immunofluorescence is well known in poultry research.

Major effects of feeding DON without an adsorbing agent on the intestinal barrier were observed in the jejunum. A significant up-regulation of CLDN5 was observed in the jejunum of the groups fed contaminated feed with or without an adsorbing agent. No significant differences were noticed in the jejunum for the mRNA expression of the other genes coding for the intestinal barrier function. The ileum on the other hand, is less susceptible to DON due to the fact that the majority of ingested DON is absorbed in the proximal parts of the small intestine [39]. However, in the group receiving DON in combination with an adsorbing agent, detrimental effects were seen in the ileum. This indicates that addition of the adsorbing agent results in a sustained presence of DON in the intestine.

The results of our study suggest that DON selectively acts on the different parts of the tight junction complex as only an up-regulation of CLDN5 was observed. A selective effect of DON has been observed *in vitro* in intestinal porcine epithelial cells and human Caco-2 cells. After 48 h exposure to DON at a concentration of 9000 ng/mL both claudin 3 and 4 showed reduced protein expression, but ZO1 and occludin were not affected [40]. The same authors also described a reduced claudin 4 expression in growing pigs after *in vivo* exposure to DON (2.85 mg DON/kg feed) for 5 weeks, using Western blot analysis and immunohistochemistry. Immunohistochemistry results showing no changes in the overall morphology of the cells, but only a decreased staining for the claudins, strengthens our hypothesis of a selective action of DON [40]. Selective action of DON on claudin-isoforms was confirmed in other more recent *in vitro* studies [41], [42]. Our study is, to our knowledge, the first one showing the effects of DON on the intestinal barrier in poultry after *in vivo* exposure to DON.

Different authors also suggest that trichothecenes may be responsible for the production of free radicals, causing damage to DNA and membranes and thus suggesting that oxidative stress may play an important role in their toxicity [43]–[47]. Up-regulation of HIF-1α often occurs in the first hours of hypoxia and, thereafter, returns to basal levels. This can be an explanation for the basal levels of HIF-1α found in the small intestine during this study. However, instead of basal expression of HIF-1α, we have observed its down-regulation in the liver of chicken, after exposure to DON or the adsorbing agent alone or in combination. As shown recently by Sparkenbaugh et al. (2011) [48], HIF-1α is up-regulated during liver injury in the initial phase of inflammation and oxidative stress, and should guarantee cell protection when the stress becomes chronic, which was not observed in our study. Furthermore, protection against hepatocyte death is related to the up-regulation of HMOX [21]. In our present study, however, hepatic HMOX was also significantly down-regulated in animals fed with adsorbent supplemented feed, contaminated with DON, or with a combination of both. In contrast, in the jejunum a significant up-regulation of HMOX was observed in the animals receiving DON contaminated feed with or without the adsorbing agent. XOR, which responds more in the chronic phase, was significantly up-regulated in the jejunum in all the animals receiving DON, but in the liver an up-regulation was observed only in the group receiving DON without an adsorbing agent. In summary, DON caused oxidative stress in the small intestine. This has previously been reported in Caco-2 cells, where DON caused a significantly increased production of malondialdehyde, a biomarker of lipid peroxidation [49]. The hepatic effects of *in vivo* exposure to 10 mg/kg DON in broiler chickens have previously been reported by Frankic et al. (2006). They observed no differences in liver content of malondialdehyde, glutathione peroxidase and total antioxidant status, which are all markers for lipid peroxidation [50]. These findings suggest a more direct genotoxic effect of DON, rather than via the oxidative pathway [51], [52].

Due to the damage to the intestinal barrier, an increased passage of non-invasive commensal bacteria may occur [53]. Both in duodenum and jejunum a significant up-regulation of TLR4 was observed during our study, which suggests inflammation, more specific due to the presence of Gram-negative bacteria [54]. In contrast, no effects on TLR2 were observed. TLR2 is more affected by the presence of Gram-positive bacteria [55].

In the last part of the small intestine, the ileum, inflammation was caused by the presence of DON in combination with the adsorbing agent. In addition, in this group all the genes coding for the tight junction complex were also up-regulated and the same trend was observed for the gene XOR, coding for oxidative stress. Along the entire length of the small intestine administration of the adsorbing agent resulted in longer villi. From our qRT-PCR results, we can conclude that it is not the adsorbing agent that causes damage as no significant differences in gene expression were seen in the group receiving control feed in combination with the adsorbing agent. The adsorbing agent is a mineral clay and seems to protect DON from degradation by the gastric fluids and intestinal enzymes in the proximal part. This may result in a higher concentration of the mycotoxin in the distal part of the small intestine when an adsorbing agent is used. Thus the binding or interaction of DON with the adsorbing agent results in a longer exposure time of the intestine to DON.

From our *in vivo* study, we can conclude that DON acts in a very specific way on the intestinal barrier in broiler chickens. Increased intestinal barrier permeability after chronic exposure to DON may lead to intestinal inflammation. The mechanism of action of DON can be different depending on the investigated target organ. The investigated mycotoxin adsorbing agent does not cause direct damage or irritation. However, feeding this clay mineral in combination with DON, may result in higher concentrations of the mycotoxin in more distal parts of the small intestine, resulting in damage of the intestinal barrier there.
